# The SWIS trial: Protocol of a pragmatic cluster randomised controlled trial of school based social work

**DOI:** 10.1371/journal.pone.0265354

**Published:** 2022-06-09

**Authors:** David Westlake, Philip Pallmann, Fiona Lugg-Widger, Donald Forrester, Stavros Petrou, Shahd Daher, Linda Adara, Laura Cook, Kim Munnery, Verity Bennett, Philip Smith, James White

**Affiliations:** 1 CASCADE, Cardiff University, Cardiff, Wales; 2 Centre for Trials Research, Cardiff University, Cardiff, Wales; 3 Nuffield Department of primary care, University of Oxford, Oxford, England, United Kingdom; Public Library of Science, UNITED KINGDOM

## Abstract

**Background:**

Child and family social workers in the UK work closely with other agencies including schools and the police, and typically they are based in local authority offices. This study will evaluate the effectiveness of placing social workers in schools (SWIS) on the need for social care interventions. SWIS was piloted in three local authorities in 2018–2020, and findings from a feasibility study of the pilots suggests SWIS may operate through three key pathways: (1) by enhancing schools’ response to safeguarding issues, (2) through increased collaboration between social workers, school staff, and parents, and (3) by improving relationships between social workers and young people.

**Methods:**

The study is a two-arm pragmatic cluster randomised controlled trial building on three feasibility studies which found SWIS to be promising. Social workers will work within secondary schools across local authorities in England. 280 mainstream secondary schools will be randomly allocated with a 1:1 ratio to SWIS or a comparison arm, which will be schools that continue as normal, without a social worker. The primary outcome will be the rate of Child Protection (Section 47) enquiries. Secondary outcomes will comprise rate of referrals to children’s social care, rate of Child in Need (Section 17) assessments, days spent in care, and educational attendance and attainment. The study also includes an economic evaluation, and an implementation and process evaluation. Social care outcomes will be measured in July 2022, and educational outcomes will be measured in July 2023. Days in care will be measured at both time points.

**Discussion:**

Findings will explore the effectiveness and cost-effectiveness of SWIS on the need for social care interventions. A final report will be published in January 2024.

**Trial registration:**

The study was registered retrospectively with the International Standard Randomised Controlled Trial Number registry on 13.11.2020 (ISRCTN90922032).

## Introduction

### Background and rationale

This study will evaluate a programme that aims to embed social workers into schools (SWIS) so that they can work more effectively with education colleagues and with children and families. Education and Children’s Social Care (CSC) have an important inter-agency relationship, and both play a vital role in keeping children safe and promoting their wellbeing. Schools have long been among the major sources of referrals to CSC in the UK, contributing the second highest proportion (18%) of all referrals in 2018/19, behind the police (29%) [1]. Yet the two agencies have different roles and priorities, and significant cultural and organisational differences add to the complexity of working effectively together. SWIS was conceived before Covid-19, but the impact of the pandemic is also putting additional pressure on both schools and social workers [2, 3]. Policymakers have been increasingly interested in finding ways to improve how education and CSC work together to respond to safeguarding concerns and protect children, and in this context these efforts are likely to intensify.

SWIS may be a promising approach to doing this. The central idea is that having a social worker linked to and based within a secondary school can have a range of benefits. In particular, to improve the service delivered to children and families, enhance interagency working, reduce risks to children and lead to better outcomes. Building on a recent set of three pilot studies [4], this project is an evaluation of the next stage of development of SWIS: a scaling up trial involving 280 schools in 21 local authorities (LAs) across England, with half of these schools receiving a social worker. Aside from the SWIS pilots, there are several examples of social workers working in schools [5, 6]. However, the scale of the SWIS programme makes this the largest example of the approach.

The three pilot studies have been central to the development of the SWIS study. They took place in Southampton, Lambeth and Stockport in 2018–20. The pilot evaluation generated qualitative and limited quantitative evidence that the intervention was positively received by schools, social care, children and families [4]. A range of benefits were evidenced, including the opportunity for social workers to do more meaningful direct work with children, help a wider range of children and families than they otherwise would, and support schools to work through safeguarding issues. The evaluation also found indications of a reduction in Child in Need and Child Protection assessments carried out. However, the strength of the evidence was limited by the quasi-experimental design, small sample size, low incidence rates of key outcomes, and short follow-up. Taken together, the qualitative and quantitative findings from the pilots prompted the Department for Education to fund this larger trial of SWIS. Delivered through What Works for Children’s Social Care, this scale-up allows us to understand the potential effectiveness of SWIS more robustly.

### Objectives

Study research questions (RQs) relate to (1) impact evaluation, (2) implementation and process evaluation, and (3) economic evaluation.

#### Impact evaluation

The primary research question is: What is the impact of SWIS in reducing rates of Section 47 enquiries (across 2 academic years, starting on 2nd September 2020 and measured 23 months later), compared to usual practice? (***RQ1***) Secondary research questions are: What is the impact of SWIS on:

- rates of referral to CSC and Section 17 assessments? (across 2 academic years, starting on 2nd September 2020 and measured 23 months later) (***RQ2***)
- the number of days children spend in care? (across 2 academic years, starting on 2nd September 2020 and measured 23 and 35 months later) (***RQ3***)
- educational attendance (recorded termly across 2 academic years, starting on 2^nd^ September 2020) (***RQ4***) and attainment (recorded June 2021 and 2022)? (***RQ5***)

#### Implementation and process evaluation

Research questions in this part of the study are: Is SWIS implemented as intended? (***RQ6***), what evidence is there for the mechanisms of change identified in the logic model? (***RQ7***), and how does SWIS impact the wider social care system? (***RQ8***).

#### Economic evaluation

The research question in this part of the study is: What is the additional cost associated with SWIS and is it justified by improvements in student outcomes? (***RQ9***).

The full study protocol is available at https://whatworks-csc.org.uk/research-project/social-workers-in-school-scale-up/.

## Materials and methods

### Trial design

The study is a pragmatic cluster randomised controlled trial with two arms (one intervention and one control group) and mainstream secondary schools as the unit of randomisation.

### Study setting

The study will be conducted in mainstream secondary schools in England, across 21 LA areas.

### Eligibility criteria

Mainstream secondary schools are places of education for children aged between 11 and 18 (Years 7 to 13). They are funded by the Government and provide a free education for children, though a number of models exist, such as academies, free schools, and faith schools. All children attending the schools are eligible for the trial. Schools may opt-out of participation in the Implementation and Process Evaluation (IPE) while remaining in the trial.

### Who will take informed consent?

Informed consent will be obtained from all individuals who take part in aspects of the IPE (e.g. surveys and interviews). Researchers will take consent, and receive signed consent forms and/ or audio recorded verbal consent from participants.

### Intervention and control conditions

The intervention physically locates social workers within schools with the aim to build better working relationships with school staff, students and families. Rather than working with students and families from a LA office base, and liaising with and providing advice to education professionals remotely, the social worker is embedded in the school. S1 Appendix shows a logic model that describes three hypothesized mechanisms of impact:

- Enhanced school response to safeguarding issues
- Increased collaboration between social worker and school staff, and parents
- Improved relationships between social worker and young people

Pathway A is based around regular, good quality, communication between the social worker and school staff. The advice and support given to school staff is hypothesized to increase staff confidence in safeguarding issues and improve the quality of school referrals. Pathway B is about working directly with families and improving relationships between social workers and parents. Pathway C is about working with children and young people directly. Frequent interactions with the social worker enable the young person to trust the social worker and to feel understood and supported. This is theorised to lead to improved school attendance and participation, better management of a young person’s risks and improved outcomes. In all three pathways, improved child and family outcomes are theorized to lead to a reduction of the number of children in care.

Following the pilot evaluations, an intervention manual has been developed (WWCSC, 2020 –available on request from programmes@whatworks-csc.org.uk). This distils the key messages from the pilots into a practical format designed to assist the 21 LAs in delivering SWIS. Its stated aim is to provide “a framework to refine the SWIS Scale-up programme and encourage more consistent and effective integration of social workers into schools”, although the need for flexibility is acknowledged. The manual offers a number of recommendations for implementers. Among them, the following relate to programme delivery:

- Social workers should be embedded within secondary schools, but can work with feeder primaries.
- Social workers linked to schools should be experienced (being in practice for at least 2 years).
- The focus should be on statutory social work with additional opportunities for “preventative” aspects, which could involve “advising staff, families and young people” and working with children who are not at the threshold for formal involvement.
- Caseloads should be managed by the SWIS team manager and be in line with LA averages, and where possible the carry-over of existing caseloads prior to the launch should be minimal. To avoid disrupting existing relationships SWIS workers are expected to take on new cases.
- Social workers should be embedded in schools as far as possible, with their own office space in the school and opportunities to integrate.
- Face to face contact should be the basis for the intervention, in order to build strong relationships with school staff, children and families.

The control group will receive service as usual. Children who are deemed by school staff to require the involvement of CSC will be referred to the LA, usually via telephone call or email to a Multi-Agency Safeguarding Hub arrangement or a referral and assessment team. Children judged by CSC to meet the threshold for involvement will be allocated a social worker as usual, but social workers will not be based in the school. This was chosen as the comparator because it is the status quo that the intervention can be distinguished from, and the system that SWIS needs to perform better than if it is to be considered worthwhile. Children who are deemed to require the involvement of CSC will continue to be eligible for this across all schools, including the control group. LAs will make case by case judgements about the best interests of children who were receiving a service prior to the trial. For example, a child on an existing child protection plan may retain their previously allocated social worker to avoid disruption, rather than transferring to the SWIS worker.

### Strategies to improve adherence to interventions

We anticipate good adherence to the intervention, as the funding for the trial intervention (i.e. the social worker) is provided to participating LAs and schools on the basis of compliance with the intervention guide supplied by the funder, and LAs have committed to delivering SWIS as described. All schools also confirmed their commitment at the outset. However, the adverse circumstances of Covid-19 may mean that some sites need to adapt SWIS, for example to accommodate social distancing or increased remote working. The IPE is designed to capture such adaptation and secondary analyses will explore the impact of varied levels of adherence.

### Outcomes

#### Primary outcome

- *Child protection (Section 47) enquiries*. Child protection (s.47) enquiries are investigations CSC carry out when they have “reasonable cause to suspect that a child who lives, or is found, in their area is suffering, or is likely to suffer, significant harm” (Children Act, 1989). This is a key point in the work of CSC; an enquiry would normally involve an assessment of the child’s needs and the ability of family members or carers to meet them. Social workers would normally interview family members, children (if they are old enough), and use information from other agencies such as schools and health. This data will be collected from LA CSC departments, based on a data sharing agreement between each LA and the research team.

Child protection (s.47) enquiry starts will be recorded as a binary (no enquiry/ enquiry started) variable. If the s.47 start date lies between and including the start date of the academic year (2nd September, 2020), and 31st July 2022, it will be coded 1, otherwise it will be coded 0. Enquiries will be recorded at the individual-level, then aggregated at the school-level and shared with the research team. This aggregated count variable will be our outcome measure.

#### Secondary outcomes

The following CSC data will be collected from LA CSC departments, based on a data sharing agreement between each LA and the research team.

- *Referrals to CSC*. Referrals are made to CSC when someone thinks a child is at risk, and schools are typically the second highest agency referrer (after the police) (Further details can be found in the version of this protocol published by the funder: Westlake et al., 2020. Referral dates will be recorded and referrals will be recorded as a binary (no referral/ referral) variable. If a referral date lies between and including the start date of the academic year (2^nd^ September, 2020), and 31st July 2022, it will be coded 1, otherwise it will be coded 0. This will be recorded at the individual-level, then aggregated at the school-level and shared with the research team. This aggregated count variable will be the outcome measure.
- *Child in Need (s*.*17) assessments*. A Child in Need assessment aims to identify the needs of a child or children within a family, and ascertain what support the family needs to meet them. Start dates for assessments will be recorded and assessments will be recorded as a binary (no assessment/ assessment) variable. If the start date for assessment lies between and including the start date of the academic year (2nd September, 2020), and 31st July 2022, it will be coded 1, otherwise it will be coded 0. These will be recorded at the individual-level, then aggregated at the school-level and shared with the research team. This aggregated count variable will be the outcome measure.
- *Days in care*. We will record the total number of days children spend in care for two time periods, 23 months and 35 months, from (and inclusive of) the start date of the academic year (2^nd^ September, 2020). We will create two variables, one that counts the number of days spent in care between and including this date and 31st July 2022, and the other that counts the number of days spent in care between and including 2^nd^ September 2020 and 31st July 2023. The inclusion of days in care at the 35 month timescale (as well as measuring it along with other CSC outcomes at 23 months) is because impact on this outcome is more likely to become clear with a longer follow-up. This will be recorded at the individual level, then aggregated at the school-level and shared with the research team. This aggregated count variable will be the outcome measure.

The following data will be collected from the Department for Education’s National Pupil Database (NPD), and will be made available to the research team in anonymised form at an individual level (identifiers will be stripped from the dataset) [7]. One application will be made to the NPD in early 2023 to request access to the following datasets:

- *Educational attendance*: *Unauthorised absences (%)*. This is the percentage of sessions (half days) that children are absent without being authorised, out of the number of sessions possible. The data are available via the NPD absence dataset. For the 2020/21 academic year, these data will be released by the NPD in March 2022, and for the 2021/22 year they will be released from October 2022 (unamended) to March 2023 (final); access will be requested for the final datasets for both years in April 2023. This variable will be defined by the number of sessions missed due to unauthorised absence per term (Autumn, Spring, Summer) out of the number of sessions possible per term.
- *Educational attainment at Key stage 4*. Key stage 4 (General Certificate of Secondary Education: GCSE) results are the key educational outcome measure for secondary school pupils. Educational attainment at Key Stage 4 (KS4) for the 2020/21 academic year will be released in April 2022, and in April 2023 for the 2021/22 academic year; access will also be requested for both years in April 2023. The following outcomes will be reported for all pupils completing GCSE exams in 2021 and 2022 or subject to equivalent grading exercise (a subset of those pupils included in the trial):
  ○ Attainment 8 –a measure of a pupil’s average grade across eight agreed GCSE subjects
  ○ English Baccalaureate Average Point Score–an agreed set of GCSE subjects (English language and literature, maths, the sciences, geography or history and a language)
  ○ % English and maths, grade 5 and above. This will be a binary variable coded 1 if the pupil achieved a level 5 or higher in both English and maths, and coded 0 otherwise

### Sample size

At the study design stage, the funder advised that a minimum of 280 mainstream schools would be available to be randomised. Assuming an average of 925 students per school, an average base rate of 12.6 s.47 enquiries per 1000 students per school year under usual practice conditions, and a between-school coefficient of variation of 0.45 within LAs (these estimates are all based on comparator school data from the three pilot studies in Lambeth, Stockport and Southampton) [4], randomising 140 mainstream schools to each group provides 90% power to detect a decrease in rates from 12.6 to 10.48 per 1000 pupils per school year (i.e. a rate ratio of 83.2%). This is using a two-sided 5% type I error level when using a Poisson regression model accounting for cluster randomisation [8].

### Recruitment

Participating LAs were chosen through a competitive tender process managed by the funder. Each chosen LA invited schools and gained the agreement from up to 16 schools to be put forward for randomisation. Recruitment of schools was completed for each LA before the list of schools was passed on to the trial statistician for randomisation. The statistician was not directly involved in the recruitment of schools. Schools were considered recruited once the LA confirmed that they have agreed to take part.

### Allocation to intervention or control groups

At the beginning of the study the research team received a list of up to 16 schools which had agreed to take part from each LA. The trial statistician performed the randomisation as described below, and the research team communicated the allocations to LAs via the funder.

### Sequence generation

Mainstream schools were allocated to SWIS or usual practice in a 1:1 ratio whilst minimising covariate imbalance within and across blocks using a balancing algorithm for cluster randomised trials with multiple blocks [9], with LAs acting as blocks. This was implemented in R version 3.6.0 [10] using code provided as supplementary material to [9]. For the first block, the standard imbalance metric (Equation 1 in [9]) was used. The allocation of subsequent blocks was conditional on blocks already allocated, using a modified imbalance metric (Equation 2 in [9]). Balancing variables were school size (total number of students enrolled in year 7 and upward) and proportion of students eligible for free school meals (FSM). Both balancing variables were weighted equally, and will be adjusted for in the final statistical analysis. The rationale for selecting these variables is as follows:

- **School size (total number of students enrolled).** The size of the school and number of students is likely to have an effect on how the social worker engages with and works within the school. In a larger school their time and resources may be spread across more students, and there may be more professionals to work with. It is reasonable to expect that this is a factor that will shape the implementation of SWIS and therefore balancing school size between groups is sensible.
- **Proportion of students eligible for free school meals.** FSM are provided for children resident in lower income households. They are designed to ensure that all children have access to adequate nutrition when they are at school. Eligibility for FSM is a reliable indicator that a child is from a low income household [11]. This is important because the work of CSC tends to be focussed on children from lower income households, and it is these children who are more likely to require a service from CSC [12].

### Data collection

#### Plans for assessment and collection of outcomes

A proforma will be supplied to the LA, who will complete and return it to the research team at each data collection time point. This process will be trialled in January 2021 prior to quarterly data transfers thereafter. Data relating to the economic evaluation (e.g. salary costs, recruitment costs and some activity time data) will also be collected in this way. Other data items required for the economic evaluation will be collected directly from individual social workers through online surveys (e.g. activity time spent implementing the intervention).

Educational outcome data will be collected from the NPD and made available anonymised at an individual-level (identifiers will be stripped from the dataset) [7]. One application will be made to the NPD in early 2023 to request the required datasets. As per the current timescales for the trial, listed below, it will be possible to access KS4 attainment and attendance data for the trial cohort and report in January 2024.

Date            Milestone

- July 2022                        Intervention ends
- January 2023            Interim report: impact on CSC outcomes, IPE and economic analysis
- April 2023            Key Stage 4 and Absence data released
- July 2023            Days in Care (35m) data collection ends
- January 2024            Final report: impact on educational and days in care outcomes

Data for the IPE will be collected primarily through online surveys and interviews. Online surveys of social workers and school staff (five surveys, one at the end of each term, all school social workers and all head teachers in intervention group invited; n = 144 social workers and circa 100–200 school staff) to gather programme-level data on implementation and attitudes and to assess how SWIS is implemented in each school (RQ6 and 8). These will be short, taking no more than fifteen minutes to complete, and administered via Qualtrics online survey platform. Interviews will form part of purposive LA case studies (n = 9; one set of 3 in terms 1–3).

### Data management

The following management plans are in place for each type of data.

#### CSC data

Data sharing agreements will be set up between LAs (Data Controller) and Cardiff University to enable sharing of data related to CSC. Data fields will be included in this data sharing agreement, data will contain no identifiers except for a school ID and/or trial allocation. A data lead will be identified at each LA, and the trial data manager will liaise with each data lead to confirm required fields. Building on our learning from the pilot, each LA will pilot data transfer during the set-up phase to ensure the data can flow and is in the correct format. These checks will be detailed in the data management plan. Once any issues are addressed in this pilot, data will be shared quarterly up to the end of the intervention (July 2022). A final data extract will be requested Summer 2023 for the days in care outcomes. Data will be reported by school year group and by month and provided to the Cardiff team in a proforma developed and agreed during the pilot. Data will be aggregated, and no individual-level data will be sent. These data will be securely transferred to Cardiff University and checked by the data manager. All data will be stored on Cardiff University servers in restricted folders available only to those on the trial team who require access. This will be detailed in the delegation log.

#### LA cost data

As per the data sharing agreement with each LA, data on costs will be sent to Cardiff using the same proforma developed for the CSC outcomes. This will be checked and prepared for onward sharing to the economics team at the University of Oxford.

#### Education data

Education attainment and attendance data will be accessed via the NPD. LAs will confirm the school identifier to the Cardiff team (Unique Reference Number, URN) to enable NPD to identify pupils in those schools. Education data for those pupils will be made available to the study team via their Secure Research Service–a remote access data safe haven (hosted by the Office for National Statistics) as per their data sharing processes [13].

#### Data processing

Trial data management staff will liaise with LA data leads and monitor proforma submissions at the specified time points. Progress will be recorded in a tracking system and all submissions will be quality checked. Automatic electronic validation checks built into the proforma will ensure data entered by LA staff complies with the specified formatting. Strict data checks will also be completed upon receipt of data collection proformas. The data manager will conduct data cleaning at each time point to ensure there are no missing/duplicated data or any outliers. Data cleaning will be a regular process and data queries will be raised with LAs if any discrepancies are found. All data queries will be logged within the tracker, and an audit trail maintained recording any changes to the data. Upon completion of data checks, the data manager will add the data to the master dataset and log the process as complete in the tracker.

### Confidentiality and data security

Personal data will be transferred to Cardiff University for the IPE. Personal data (name and contact details) of social workers, school staff and children will be transferred to Cardiff University by LAs for those who have agreed for this information to be sent. Study information will be made available to IPE participants prior to participation. A risk assessment has been signed off to mitigate any potential risks regarding confidentiality and identification while processing these data, and personal data will be stored on secure university network servers, with access limited to members of the research team. No personal data will be processed by Cardiff University for the impact evaluation or economic workstream. All data will be stored in a secure manner and processed in accordance with data protection legislation (in accordance to GDPR and UK DPA 18) and Good Clinical Practice (GCP).

**Fig 1:**
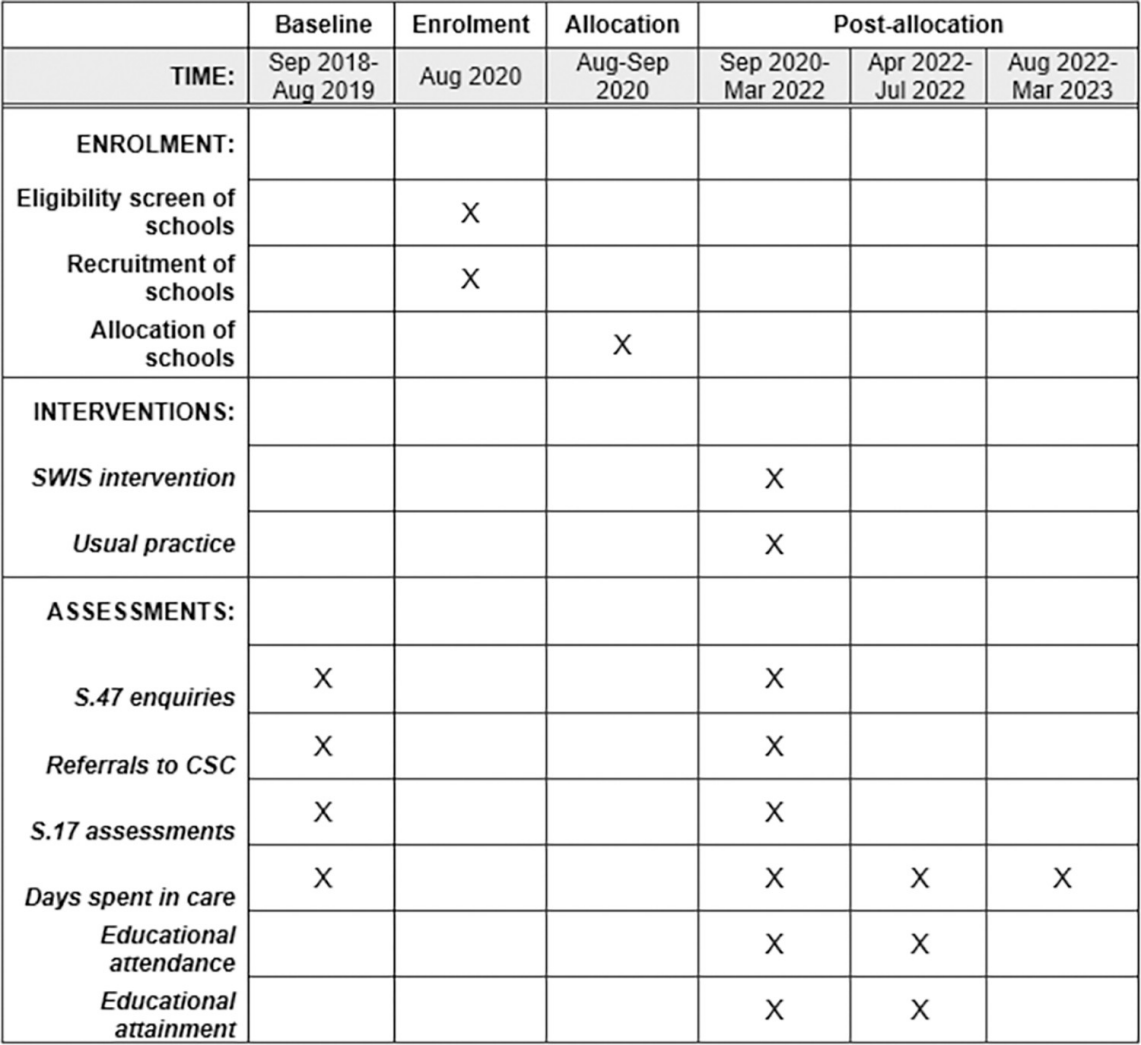
SPIRIT checklist.

The elements and timing of data collection are shown in Fig 1 below.

### Statistical methods

#### Statistical methods for primary and secondary outcomes

All primary analyses will be ‘intention to treat’ i.e. schools will be analysed in the groups to which they were randomised, regardless of adherence to the intervention. Missing outcome data will not be replaced. For the primary outcome analysis, a quasi-Poisson regression model will be fitted with fixed effects for LAs and cluster robust standard errors reflecting the clustering structure (schools within LAs) to compare rates of s.47 enquiries at 23 months by arm, using the number of students per school as the exposure scaling variable, and s.47 enquiries for 2018/19 (baseline) and % eligible for FSM as fixed-effect covariates. Using a quasi-likelihood model accounts for possible over- or underdispersion i.e. variance in the data that is higher or lower than expected under the standard Poisson model. Allocation will be included in the model as a fixed effect, and the intervention effect (model coefficients transformed into rate ratios) will be presented as point estimate with two-sided 95% confidence interval (CI) and p-value. If the estimated rate is lower in the SWIS arm than in the comparator arm and the 95% CI around the estimated rate ratio excludes the null effect (rate ratio = 1), effectiveness of SWIS at the 5% level will be concluded.

Secondary outcomes will be analysed in a similar way as the primary outcome, by fitting regression models with cluster robust standard errors depending on the type of outcome: quasi-Poisson for rates (referrals to CSC, s.17 assessments) and linear for continuous variables (days in care, educational attendance and attainment). For the school-level variables (referrals to CSC, s.17 assessments, days in care) we will include the same fixed-effect covariates in the model as for the primary outcome (allocation, LA, baseline outcome from 2018/19, % eligible for FSM) and also use the number of students per school as the exposure scaling variable. For the student-level variables (educational attendance and attainment) we will additionally include gender and ethnicity as covariates, and the baseline outcomes used will be 2018/19 attendance (for attendance) and KS4 results (for attainment). The p-values generated from the secondary outcome analyses will be adjusted for multiplicity using Hochberg’s step-up procedure.

#### Other secondary analyses

A secondary analysis will use per-term (or per-month, if available) outcome data and include term (or month) as an additional covariate in the model to explore potential implementation effects and/or seasonality. Another secondary analysis will assess the hypothesised mediators of change outlined in logic model (S1 Appendix) at the 23-month follow-up by fitting an interaction term between allocation and category of implementation fidelity (a Gold, Silver, Bronze categorisation will be developed based on a re-analysis of pilot data and other insights from the IPE). Any other subgroup analyses (e.g. by age group) will be agreed with service user input. The p-values generated from these secondary and subgroup analyses will also be adjusted for multiplicity using Hochberg’s step-up procedure. We will fit a two-level mixed-effects model with random LA effects as an additional robustness analysis.

Missing outcome data will not be replaced in the primary analysis, but we will perform null imputation for missing covariates, where we will replace missing values with 0, and create a dummy indicator for the covariate coded 1 if the value was missing, and 0 otherwise, and include the dummy in the regression. If more than 5% of outcome data are missing, we will consider repeating the primary analysis after multiple imputation. To assess the impact of non-compliance (if present), we will exclude intervention arm schools that did not adopt the intervention at all and then repeat the primary analysis, and we will also perform a complier average causal effect (CACE) analysis. Statistical code and anonymised data can be provided upon reasonable request, in line with Centre for Trials Research policy.

### Ethical considerations

The study has ethical approval from School of Social Sciences Research Ethics Committee of Cardiff University (Ref: SREC/3865, approval date: 26.08.2020). Written, informed consent to participate will be obtained from all participants. Due to the use of remote methods, and in case consent forms are not returned, verbal consent will be audio recorded in interviews with professionals in addition to written consent.

If interviewees say anything that makes the researcher concerned about harm to the participant or another person, then they have a duty to take appropriate action. In the first instance, usually this would involve discussing the concern with the Chief Investigator or a co-investigator. Depending on the nature of the harm, referrals to agencies may be appropriate, for example a referral to the LA CSC may be deemed necessary if a child was thought to be at risk.

### Trial status

LAs are classed as ‘recruited’ once they have signed the data sharing agreement. Recruitment began (data sharing agreements sent for signature) on 22 October 2020. Recruitment was completed in February 2021. The trial was extended twice by the funder, first in August 2021 and then in January 2022 (see below) Amended data sharing agreements to cover the extended period were circulated to LAs for signature n September 2021 and March 2022.

## Discussion

The SWIS trial is the largest and most rigorous study of school based social work in the UK, and will be a significant addition to the international evidence base. It will contribute to our understanding of how multi-agency responses may help protect children and reduce the need for CSC involvement, and investigate whether working in this way has an impact on educational outcomes for vulnerable children. However, this is a time of great disruption as a result of the Covid-19 pandemic, and our findings will need to be considered in light of this.

Covid-19 is likely to present ongoing challenges for both the implementation of SWIS and our efforts to study it. As the implementation guide states, the intention is that “face to face contact should be the basis for the intervention” but this may simply not be possible. Although most schools remained open for ‘vulnerable’ children at the start of 2021 [14], many children in this category were not attending school [15]. Schools involved in the trial also introduced measures to make schools safer and reduce the chances of disease transmission, including limiting numbers of staff on site. This will almost certainly affect the way social workers are able to engage with children, families and professionals.

Moreover, some of the outcome indicators we intend to use may not be comparable to the same measures in previous years. Attendance and attainment measures, for example, appear especially incomparable with pre-pandemic times. For example, whether or not an absence is unauthorised will be more difficult to record, and Key Stage 4 examinations will be replaced with teacher assessed grades in 2021 [16].

At a broader level, the amount of disruption implementers face will make the results of our between groups analysis difficult to interpret. Covid-19 has increased the risk of misleading results, as (1) the implementation of SWIS is likely to be more variable and (2) exogenous factors may override the impact of the intervention. The IPE is designed to insure against these risks, and in this context the secondary and exploratory analyses that it will feed into will gain importance. It would be easier to address these challenges if the trial ran for more than a single academic year. However, as it is linked to a policy initiative the study is limited to the timescale set out by the funder.

### Amendments to the study

The study was extended twice by the funder, first in August 2021, and then in January 2022.This protocol was amended to accommodate the new timeline. This document reflects the amended study design. Both the original protocol (version 1) and the revised protocol (versions 2 and 3) can be found on the OSF. Key amendments made in the updated protocols are:

- Timing of analysis and reporting updated to reflect the extended intervention period (e.g. meaning that the main impact analysis will now be published in January 2023 rather than 2021, and the follow-up analysis and final report will now be published in January 2024 instead of June 2022).
- Changes to the data collection activities in the IPE, to reflect the fact that all case studies were forced online by the pandemic and conducted remotely. This meant that as of September 2021 no observations or child interviews have taken place, and these are now scheduled for terms 4 and 5.
- Additional data collection activities in the three extra terms, e.g. interviews with key decision makers in LA ‘front door’ teams.
- The use of the 2018/19 school year as a baseline, rather than the 2019/20 school year as originally planned. This is because the 2018/19 year is the most recent year not affected by the Covid-19 pandemic and therefore a more realistic baseline to use.
- Changes to the economic evaluation to reflect discounting to costs beyond 12 months from the start of the intervention, to present values using nationally recommended discount rates.

### Dissemination plans

We are contracted to report the findings in reports that will be published open access by the funder at https://whatworks-csc.org.uk/. We will also disseminate the study widely by other means, using in-person methods (e.g. conference presentations and invited talks), electronic methods (e.g. taking part in a podcast and online videos produced by the funder, publishing blogs on the university webpages) and by publishing in academic journals.

## Supporting information

S1 Appendix Logic model showing hypothesized mechansims of action underpinnning SWIS. (DOCX)

S2 Appendix Study flow diagram. (DOCX)

## Abbreviations

CSC: Children’s Social Care
SWIS: Social Workers in Schools
IPE: Implementation and Process Evaluation
GCSE: General Certificate of Secondary Education
KS: Key Stage
FSM: Free School Meals
DSA: Data Sharing Agreement
NPD: National Pupil Database
LA: Local Authority
CI: Confidence Interval
URN: Unique Reference Number for schools
