# Supplementary figures and images for: The SWIS trial: Protocol of a pragmatic cluster randomised controlled trial of school based social work
Source: PLoS One. 2022 Jun 9;17(6):e0265354. doi: 10.1371/journal.pone.0265354 (PMC9182565; doi:10.1371/journal.pone.0265354)

**Appendix 1. Logic model showing hypothesized mechansims of action underpinnning SWIS**


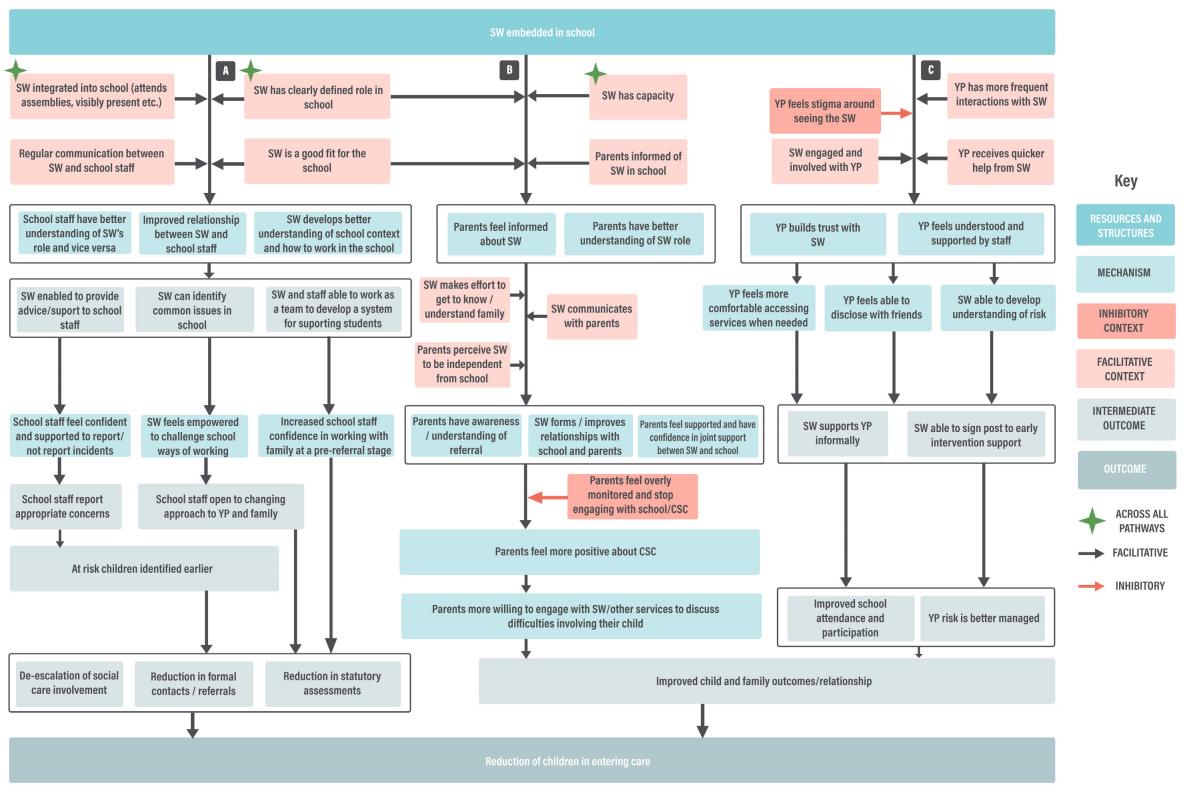

Supplement: S1 Appendix — (DOCX) [file pone.0265354.s001.docx]
