# Supplementary material for: The SWIS trial: Protocol of a pragmatic cluster randomised controlled trial of school based social work
Source: PLoS One. 2022 Jun 9;17(6):e0265354. doi: 10.1371/journal.pone.0265354 (PMC9182565; doi:10.1371/journal.pone.0265354)
Supplement: S2 Appendix — (DOCX) [file pone.0265354.s002.docx]

**Appendix 2. Study flow diagram**

LA’s submit lists of nominated schools

Basic data on schools collated and used for balancing procedure

LA’s and schools informed of randomisation outcome

SWIS intervention schools

Control group (usual practice) schools

Final reporting of additional outcomes Educational attendance recorded termly up to month 23; Educational attainment recorded at 10 & 22 months; days in care at 35 months

23 months assessment of CSC outcomes; IPE evaluation findings reported; Economic evaluation findings reported

Intervention delivery begins

Intervention delivery ends

IPE evaluation

Termly activities:

Online survey of social workers and school staff (every term)

Case study visits to 3 LA’s including interviews with children, social workers and school staff (term 1-3)

Interviews with children (term 4-5)

Retrospective analysis in terms 5:

Analysis of referral data from all sources

Interviews with other key LA stakeholders

Retrospective interviews with social workers and school staff

Schools randomised using 1:1 ratio

23 months assessment of CSC outcomes; Economic evaluation findings reported

Final reporting of additional outcomes Educational attendance recorded termly up to month 23; Educational attainment recorded at 10 & 22 months; days in care at 35 months
